# Supplementary material for: A Technology-Driven, Healthcare-Based Intervention to Improve Family Beverage Choices: Results from a Pilot Randomized Trial in the United States
Source: Nutrients. 2023 Apr 29;15(9):2141. doi: 10.3390/nu15092141 (PMC10181264; doi:10.3390/nu15092141)

Supplement to: A technology-driven, clinic-based intervention to reduce child sugary beverage consumption: results from a pilot randomized trial

S1: Detailed Description of Intervention Components

S2: Details of Intervention Fidelity & Acceptability

S1: Detailed Description of Intervention Components

Water Promotion Toolkit: The toolkit included dishwasher-safe, BPA-free water bottles (2 for children, 1 for adults, per family), BPA-free infusers that could be filled with fruits, vegetables or herbs and inserted into the bottles to flavor water, a sheet of kid-friendly, dishwasher-safe stickers that could be used by children to decorate and personalize their water bottles, a children's book about drinking water (Potter the Otter, a Tale about Water)[1], and a pamphlet welcoming families to the Ready, Set, Gulp! Program and instructing parents on how to use the toolkit. The pamphlet also had step-by-step instructions on how to access remaining intervention components, beginning with a QR code to scan for watching the video (described below). The toolkit was provided to families by their pediatrician in a cloth carrying bag bearing the study logo.

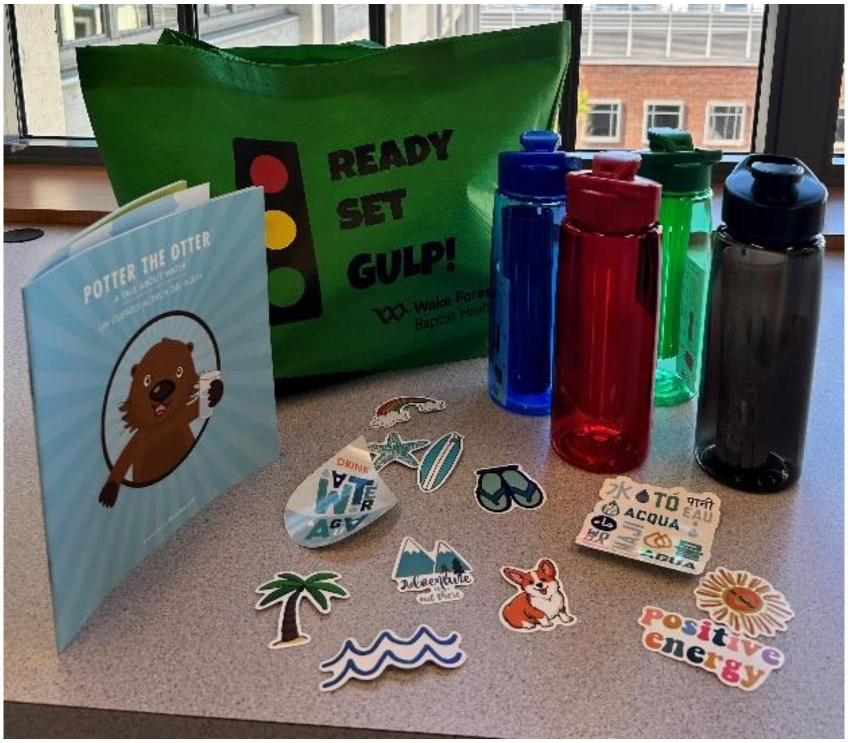

1. Grummon AH, Sokol RL, Goodman D, et al. Storybooks About Healthy Beverage Consumption: Effects in an Online Randomized Experiment With Parents. *Am J Prev Med.* 2022;62(2):183-192.

Get in the Zero Zone! Video: The video was developed using parental focus groups in partnership with health communications faculty from Wake Forest University. It featured a “reality TV” narrative style where three real-world families were recruited and followed for a one-week challenge to eliminate sugary drinks and consume more water. The 5-minute video included information about health consequences of sugary drinks, educated parents about the sugar content of common drink types, suggested tips and tools for getting the family to drink more water, and featured the idea of engaging the family in goal-setting and tracking beverage intake, and rewarding positive behavior changes.

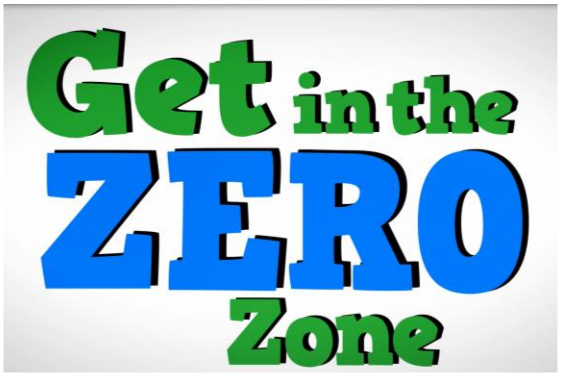

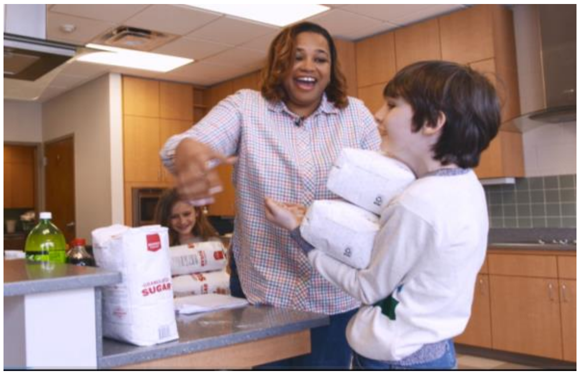

Video can be accessed here: https://youtu.be/W7Wyf_nejG8

<u>Ready, Set, Gulp Mobile Phone Application</u>: This app was developed based on parental interview feedback, in partnership with the Wake Forest App Development Team over a one year period. An initial prototype was further refined by conducting 1-week usability tests with 9 parents. The app featured a racetrack theme and prominently featured the red/yellow/green (stoplight) designation to provide easy visual cues for the healthfulness of different drink types and behaviors. Within the app, each family member could choose and customize a vehicle avatar to represent themselves. Individuals could then earn points by engaging with various app functions, and advance their vehicles toward a finish line. At the end of each week, the user with the most points within each family was awarded a winner badge, and all users were returned to the starting line. The app included a journaling function to track what and how much each user drank each day (parents were instructed to do this on behalf of children under 5 and to assist their older children). It also featured a “Pop Quiz” where users could earn points for correctly answering trivia questions about sugary drinks, water and general health and nutrition. There was a goal setting function where each user could create targets for SSB, FJ and water consumption, and a dashboard where users could see their daily consumption patterns and whether or not goals were being met. The “Flavor Finder” section of the app allowed users to search for, create and rate recipes for different types of flavor infused waters, and the “Sugar Translator” section allowed users to see how much sugar was in their drinks by “translating” any particular drink choice and volume into an equivalent amount of a common dessert item (e.g. they could see that 1 12-ounce can of cola would have the same amount of sugar as 2 scoops of vanilla ice cream, or 1.5 pieces of birthday cake). An overview of the app can be viewed here: https://youtu.be/ZCOxt2SDGK0

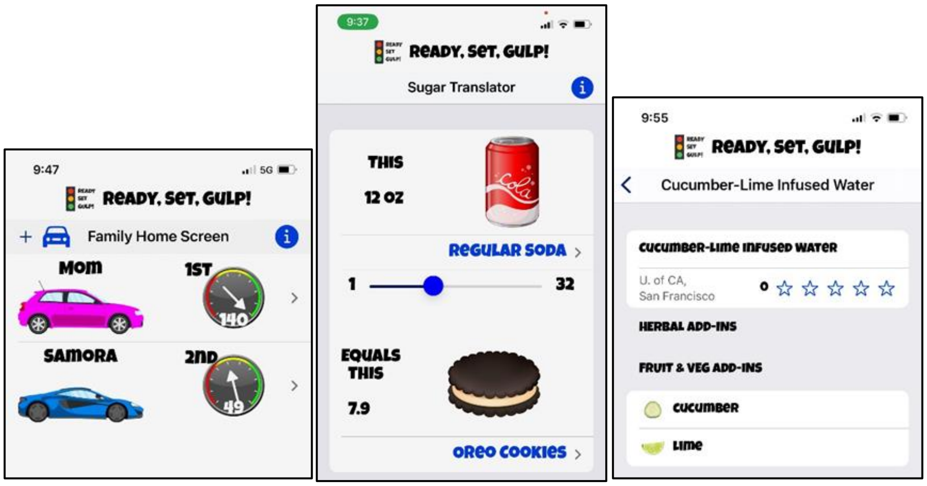

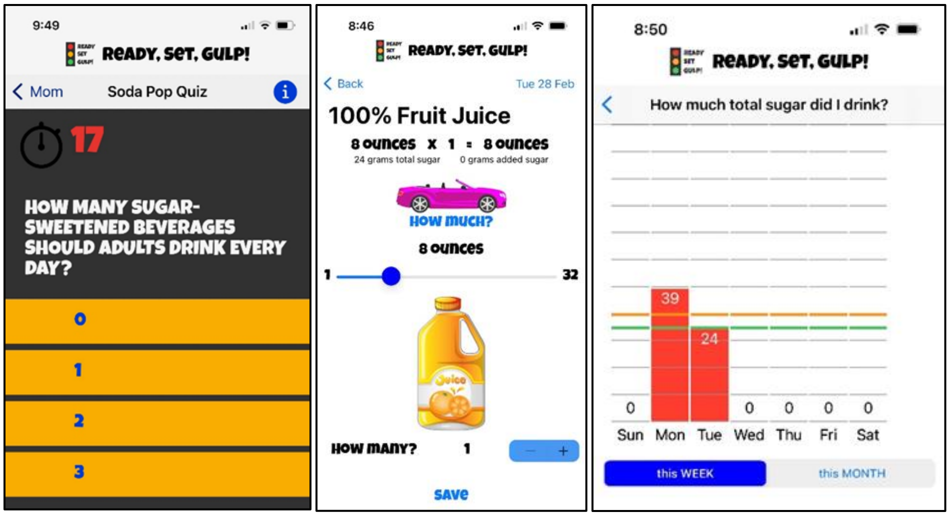

<u>Interactive Voice Response (IVR) Calls</u>: This call series consisted of 14 interactive voice response (IVR) phone calls and was developed in partnership with the Wake Forest Digital Communications group (DComm). The calls were made to parents using the Twilio platform, linked to REDCap and were automatically scheduled over a 6 month period beginning when parents completed watching the video. Calls were initially spaced weekly and scheduled for Saturdays at noon, but gradually spaced at wider intervals over follow-up, as detailed in the schedule below. Each call lasted 3-5 minutes and used an AI-assisted computerized voice to speak to parents and record their responses. All calls were made to the parent participant at a phone number they provided. Each call started by introducing the Ready, Set, Gulp! study as the caller and asking if it was a good time for the parent to speak. If so, the AI voice proceeded with a pre-determined script, which differed based on the call number. All calls included an opportunity for parents to review goals around family behavior change and set new goals for water consumption, and fruit juice or sugary drink consumption. The topics covered in the more didactic portion of the call were chosen to address environmental and psychosocial barriers to behavior change that parents had identified in the formative phases of intervention development.

**Call #**	**Week**	**Content**
1	1	Introduction, Importance, Establish Baseline, Set initial goals
2	2	Review & Renew Goals, **Tap water safety**
3	3	Review & Renew Goals, **Kids don’t like plain water**
4	4	Review & Renew Goals, **Kids act out when SSB removed**
5	5	Review & Renew Goals, **Tap water saves $ and cuts down on sugar**
6	6	Review & Renew Goals, **Tap water tastes funny**
7	7	Review & Renew Goals, **Getting other family members on board**
8	8	Review & Renew Goals, **Children getting SSB from outside source**
9	10	Review & Renew Goals, **Get too busy and forget to drink H2O**
10	12	Review & Renew Goals, **Not feeling motivated to change**
11	14	Review & Renew Goals, **More tips to get kids excited about H2O**
12	16	Review & Renew Goals, **More tips to cut back on SSB & FJ**
13	20	Review & Renew Goals, **Shopping & label reading**
14	24	Review & Renew Goals, **Sustaining changes moving forward**

S2: Details of Intervention Fidelity & Acceptability

We collected objective and subjective data on intervention acceptability and fidelity. Fidelity was monitored using REDCap which allowed us to track video viewing and completion of all IVR calls. We tracked app downloads using individualized codes. Subjective feedback on the video was collected using an online survey via REDCap which took place immediately after the video was viewed. All 29 parents who viewed the video completed this survey. After 6-month data collection was completed, parents remaining in the intervention group (n=24) were scheduled for and completed a telephone-based exit interview, during which a series of survey items were verbally administered by a research team member. Survey items asked about use and preferences for each of the intervention components. Those responses are summarized here, separated by intervention component.

Water Promotion Toolkit

Survey Question	Response Options	N (%) or Mean (sd)
Over the last 6 months, how often did you use one of the water bottles that was provided to drink water?	Never<br>Rarely<br>About once or twice a week<br>Most Days<br>Every Day	3 (12.5%)<br>2 (8.3%)<br>5 (20.8%)<br>6 (25%)<br>8 (33.3%)
Over the last six months, about how often did another adult in your home use one of the water bottles that was provided to drink water?	Never<br>Rarely<br>About once or twice a week<br>Most Days<br>Every Day<br>Not Applicable (no other adults)	5 (20.8%)<br>1 (4.2%)<br>1 (4.2%)<br>8 (33.3%)<br>4 (16.7%)<br>5 (20.8%)
Over the last six months, about how often did your child use one of the water bottles that was provided to drink water?	Never<br>Rarely<br>About once or twice a week<br>Most Days<br>Every Day	4 (16.7%)<br>0 (0%)<br>5 (20.8%)<br>6 (25%)<br>9 (37.5%)
Over the last six months, about how often did another child in your home besides [child's name in intervention] use one of the water bottles that was provided to drink water?	Never<br>Rarely<br>About once or twice a week<br>Most Days<br>Every Day<br>Not Applicable (no other children)	5 (20.8%)<br>1 (4.2%)<br>6 (25%)<br>3 (12.5%)<br>6 (25%)<br>3 (12.5%)
How often did you or people in your family put additional flavors in your water using the infusers	Never<br>Rarely<br>About once or twice a week	6 (25%)<br>3 (12.5%)<br>8 (33.3%)

(either built into the water bottle or added in for kids bottles)?	Most Days<br>Every Day	7 (29.2%)<br>0 (0%)
I drank more water because of the water bottles	Not Applicable (did not use)<br>Strongly Disagree<br>Disagree<br>Neither<br>Agree<br>Strongly Agree	2 (8.3%)<br>0 (0%)<br>3 (12.5%)<br>8 (33.3%)<br>8 (33.3%)<br>3 (12.5%)
My child drank more water because of the water bottles	Not Applicable (did not use)<br>Strongly Disagree<br>Disagree<br>Neither<br>Agree<br>Strongly Agree	0 (0%)<br>0 (0%)<br>2 (8.3%)<br>4 (16.7%)<br>9 (37.5%)<br>9 (37.5%)
Using the infusers helped water taste better to me	Not Applicable (did not use)<br>Strongly Disagree<br>Disagree<br>Neither<br>Agree<br>Strongly Agree	3 (12.5%)<br>2 (8.3%)<br>3 (12.5%)<br>3 (12.5%)<br>9 (37.5%)<br>4 (16.7%)
Using the infusers helped water taste better to my child	Not Applicable (did not use)<br>Strongly Disagree<br>Disagree<br>Neither<br>Agree<br>Strongly Agree	3 (12.5%)<br>2 (8.3%)<br>2 (8.3%)<br>6 (25%)<br>7 (29.2%)<br>4 (16.7%)
How often did your child read the "Potter the Otter" book that was included in the goodie bag (including times (s)he read it alone OR when you helped him/her or read to him/her)?	Never<br>Only once or twice<br>Many times<br>I don't know	0 (0%)<br>20 (83.3%)<br>3 (12.5%)<br>1 (4.2%)
Mean rating (1-10 scale; 1 is worst, 10 is best) for **water bottles** component of toolkit	Mean parental rating	8.9
Mean rating (1-10 scale; 1 is worst, 10 is best) for **fruit infusers** component of toolkit	Mean parental rating	7.0
Mean rating (1-10 scale; 1 is worst, 10 is best) for **stickers** component of toolkit	Mean parental rating	9.0
Mean rating (1-10 scale; 1 is worst, 10 is best) for **Potter the Otter book** component of toolkit	Mean parental rating	8.2
Mean rating (1-10 scale; 1 is worst, 10 is best) for g**oodie bag with "Ready Set Gulp!" logo** component of toolkit	Mean parental rating	8.1

Video

**Survey Question**	**Response Options**	**N (%) or Mean (sd)**
Thinking about the "Get in the Zero Zone!" Video that you just watched, please indicate how much you DISAGREE or AGREE with each of the following	Strongly Disagree<br>Disagree<br>Neither<br>Agree<br>Strongly Agree	0 (0%)<br>0 (0%)<br>2 (6.9%)<br>9 (31%)<br>18 (62.1%)

statements: “I enjoyed watching this video”		
Thinking about the "Get in the Zero Zone!" Video that you just watched, please indicate how much you DISAGREE or AGREE with each of the following statements: ”I learned more about what kids are supposed to drink by watching this video”	Strongly Disagree<br>Disagree<br>Neither<br>Agree<br>Strongly Agree	0 (0%)<br>2 (6.9%)<br>1 (3.4%)<br>11 (37.9%)<br>15 (51.7%)
Thinking about the "Get in the Zero Zone!" Video that you just watched, please indicate how much you DISAGREE or AGREE with each of the following statements: “I understood most of what was said in this video”	Strongly Disagree<br>Disagree<br>Neither<br>Agree<br>Strongly Agree	0 (0%)<br>0 (0%)<br>0 (0%)<br>6 (20.7%)<br>23 (79.3%)
Thinking about the "Get in the Zero Zone!" Video that you just watched, please indicate how much you DISAGREE or AGREE with each of the following statements: “I can identify with or relate to the families in this video”	Strongly Disagree<br>Disagree<br>Neither<br>Agree<br>Strongly Agree	0 (0%)<br>0 (0%)<br>2 (6.9%)<br>14 (48.3%)<br>13 (44.8%)
Thinking about the "Get in the Zero Zone!" Video that you just watched, please indicate how much you DISAGREE or AGREE with each of the following statements: ”I would want to watch this video more than once”	Strongly Disagree<br>Disagree<br>Neither<br>Agree<br>Strongly Agree	1 (3.4%)<br>3 (10.3%)<br>5 (17.2%)<br>10 (34.5%)<br>10 (34.5%)
Thinking about the "Get in the Zero Zone!" Video that you just watched, please indicate how much you DISAGREE or AGREE with each of the following statements; “I would want to share this video with my family or friends”	Strongly Disagree<br>Disagree<br>Neither<br>Agree<br>Strongly Agree	0 (0%)<br>0 (0%)<br>4 (13.8%)<br>11 (37.9%)<br>14 (48.3%)
Thinking about the "Get in the Zero Zone!" Video that you just watched, please indicate how much you DISAGREE or AGREE with each of the following statements: “My kids would enjoy watching this video”	Strongly Disagree<br>Disagree<br>Neither<br>Agree<br>Strongly Agree	0 (0%)<br>2 (6.9%)<br>9 (31%)<br>10 (34.5%)<br>8 (27.6%)
Thinking about the length of the video - which of the following do you most agree with. (pick one)	Just about right<br>Too short<br>Too long	27 (93.1%)<br>1 (3.4%)<br>1 (3.4%)

Mobile Phone App

Survey Question	Response Options	N (%) or Mean (sd)
Did you or your family download and use the Ready, Set, Gulp app at least once during the last six months?	Yes<br>No	22 (91.7%)<br>2 (8.3%)
Thinking about the "Ready, Set, Gulp!" App, please indicate how much you agree or disagree with each of the following statements: "I think that I would like to use this app frequently"	Strongly Disagree<br>Disagree<br>Neither<br>Agree<br>Strongly Agree	2 (9.1%)<br>2 (18.2%)<br>5 (22.7%)<br>9 (40.9%)<br>2 (9.1%)
Thinking about the "Ready, Set, Gulp!" App, please indicate how much you agree or disagree with each of the following statements: "I found the app too complex"	Strongly Disagree<br>Disagree<br>Neither<br>Agree<br>Strongly Agree	4 (18.2%)<br>14 (63.6%)<br>4 (18.2%)<br>0 (0%)<br>0 (0%)
Thinking about the "Ready, Set, Gulp!" App, please indicate how much you agree or disagree with each of the following statements: "I thought the app was easy to use"	Strongly Disagree<br>Disagree<br>Neither<br>Agree<br>Strongly Agree	0 (0%)<br>1 (4.5%)<br>3 (13.6%)<br>13 (59.1%)<br>5 (22.7%)
Thinking about the "Ready, Set, Gulp!" App, please indicate how much you agree or disagree with each of the following statements: "I think that I would need the support of a technical person to be able to use this app"	Strongly Disagree<br>Disagree<br>Neither<br>Agree<br>Strongly Agree	8 (36.4%)<br>13 (59.1%)<br>1 (4.5%)<br>0 (0%)<br>0 (0%)
Thinking about the "Ready, Set, Gulp!" App, please indicate how much you agree or disagree with each of the following statements: "I found the various functions in this app worked well together"	Strongly Disagree<br>Disagree<br>Neither<br>Agree<br>Strongly Agree	0 (0%)<br>0 (0%)<br>4 (18.2%)<br>13 (59.1%)<br>5 (22.7%)
Thinking about the "Ready, Set, Gulp!" App, please indicate how much you agree or disagree with each of the following statements: "I thought there were too many different things going on in this app"	Strongly Disagree<br>Disagree<br>Neither<br>Agree<br>Strongly Agree	4 (18.2%)<br>12 (54.5%)<br>4 (18.2%)<br>1 (4.5%)<br>1 (4.5%)
Thinking about the "Ready, Set, Gulp!" App, please indicate how much you agree or disagree with each of the following statements: "I would imagine that most people would learn to use this app very quickly'	Strongly Disagree<br>Disagree<br>Neither<br>Agree<br>Strongly Agree	0 (0%)<br>1 (4.5%)<br>3 (13.6%)<br>13 (59.1%)<br>5 (22.7%)
Thinking about the "Ready, Set, Gulp!" App, please indicate how much you agree or disagree with each of the following statements: "I found the app very difficult or clunky to use"	Strongly Disagree<br>Disagree<br>Neither<br>Agree<br>Strongly Agree	6 (27.3%)<br>13 (59.1%)<br>3 (13.6%)<br>0 (0%)<br>0 (0%)
Thinking about the "Ready, Set, Gulp!" App, please indicate how much you agree or disagree with each of the following statements:	Strongly Disagree<br>Disagree<br>Neither<br>Agree	0 (0%)<br>3 (13.6%)<br>2 (9.1%)<br>13 (59.1%)

"I felt very confident using the app"	Strongly Agree	4 (18.2%)
Thinking about the "Ready, Set, Gulp!" App, please indicate how much you agree or disagree with each of the following statements: "I needed to learn a lot of things before I could get going with this app	Strongly Disagree<br>Disagree<br>Neither<br>Agree<br>Strongly Agree	7 (31.8%)<br>12 (54.5%)<br>3 (13.6%)<br>0 (0%)<br>0 (0%)
Mean rating (1-10 scale; 1 is worst, 10 is best) for **Graphics/pictures** in the app?	Mean parental rating	8.4
Mean rating (1-10 scale; 1 is worst, 10 is best) for **Account setup process (adding your information, selecting your avatar)** in the app	Mean parental rating	9.0
Mean rating (1-10 scale; 1 is worst, 10 is best) for **Goal-setting process** in the app	Mean parental rating	8.8
Mean rating (1-10 scale; 1 is worst, 10 is best) for **Home screen with car avatars on race track** in the app	Mean parental rating	8.5
Mean rating (1-10 scale; 1 is worst, 10 is best) for **Journal function (recording what you had to drink)** in the app	Mean parental rating	8.1
Mean rating (1-10 scale; 1 is worst, 10 is best) for **Pop Quiz function (answering trivia questions)** in the app	Mean parental rating	8.5
Mean rating (1-10 scale; 1 is worst, 10 is best) for **Sugar Translator function (how much sugar is in different drinks compared to common dessert foods)** in the app	Mean parental rating	9.1
Mean rating (1-10 scale; 1 is worst, 10 is best) for **Flavor Finder function (making and rating recipes for flavored water)** in the app	Mean parental rating	8.6
Mean rating (1-10 scale; 1 is worst, 10 is best) for **Personal Dashboard (graphs where you can track your progress over time)** in the app	Mean parental rating	8.9
Mean rating (1-10 scale; 1 is worst, 10 is best) for **Notifications and Reminders** in the app	Mean parental rating	8.5
Mean rating (1-10 scale; 1 is worst, 10 is best) for **Red Yellow Green color scheme** in the app	Mean parental rating	9.4
While using the app over the past few months, did you or your family have any of the following problems?	App crashing<br>App freezing<br>App too slow to load<br>App not storing data<br>Drink not found in journal<br>Did not understand information	2 (9.1%)<br>6 (27.3%)<br>0 (0%)<br>0 (0%)<br>0 (0%)<br>1 (4.5%)

	Images/Text too small<br>None of the above	0 (0%)<br>15 (68.2%)
For each of the following statements, please indicate whether you agree or disagree: "Because of the app, I drank more water"	Strongly Disagree<br>Disagree<br>Neither<br>Agree<br>Strongly Agree	1 (4.5%)<br>5 (22.7%)<br>8 (34.6%)<br>6 (27.3%)<br>2 (9.1%)
For each of the following statements, please indicate whether you agree or disagree: "Because of the app, I drank less juice or sugar-sweetened beverages"	Strongly Disagree<br>Disagree<br>Neither<br>Agree<br>Strongly Agree	1 (4.5%)<br>5 (22.7%)<br>5 (22.7%)<br>10 (45.5%)<br>1 (4.5%)
For each of the following statements, please indicate whether you agree or disagree: "I learned new information about water by using the app"	Strongly Disagree<br>Disagree<br>Neither<br>Agree<br>Strongly Agree	1 (4.5%)<br>3 (13.6%)<br>5 (22.7%)<br>12 (54.5%)<br>1 (4.5%)
For each of the following statements, please indicate whether you agree or disagree: "I learned new information about sugary drinks by using the app"	Strongly Disagree<br>Disagree<br>Neither<br>Agree<br>Strongly Agree	1 (4.5%)<br>2 (9.1%)<br>5 (22.7%)<br>12 (54.5%)<br>2 (9.1%)
For each of the following statements, please indicate whether you agree or disagree: "I learned more about what my kids are supposed to drink by using the app"	Strongly Disagree<br>Disagree<br>Neither<br>Agree<br>Strongly Agree	0 (0%)<br>2 (9.1%)<br>5 (22.7%)<br>11 (50%)<br>4 (18.2%)
For each of the following statements, please indicate whether you agree or disagree: "The app motivated me to want to change my own drink choices"	Strongly Disagree<br>Disagree<br>Neither<br>Agree<br>Strongly Agree	1 (4.5%)<br>5 (22.7%)<br>7 (31.8%)<br>7 (31.8%)<br>2 (9.1%)
For each of the following statements, please indicate whether you agree or disagree: "The app motivated me to want to change my kids' drink choices"	Strongly Disagree<br>Disagree<br>Neither<br>Agree<br>Strongly Agree	0 (0%)<br>5 (22.7%)<br>5 (22.7%)<br>7 (31.8%)<br>5 (22.7%)
For each of the following statements, please indicate whether you agree or disagree: "I tried out new flavorings for my drinking water because of the app"	Strongly Disagree<br>Disagree<br>Neither<br>Agree<br>Strongly Agree	1 (4.5%)<br>7 (31.8%)<br>6 (27.3%)<br>6 (27.3%)<br>2 (9.1%)
For each of the following statements, please indicate whether you agree or disagree: "After using the app, I am more confident about the safety of drinking tap water"	Strongly Disagree<br>Disagree<br>Neither<br>Agree<br>Strongly Agree	2 (9.1%)<br>4 (18.2%)<br>5 (22.7%)<br>9 (40.9%)<br>2 (9.1%)
How often did your children use the app?	Never<br>A few times but not every day<br>Once a day<br>More than once a day<br>N/A	13 (59.1%)<br>6 (27.3%)<br>0 (0%)<br>0 (0%)<br>3 (13.6%)
For each of the following statements, please indicate whether you agree or disagree: "Because of the app, my kids drank more water"	Strongly Disagree<br>Disagree<br>Neither<br>Agree<br>Strongly Agree	0 (0%)<br>2 (22.2%)<br>5 (55.6%)<br>1 (11.1%)<br>1 (11.1%)

For each of the following statements, please indicate whether you agree or disagree: “Because of the app, my kids drank less juice or sugar-sweetened beverages”	Strongly Disagree<br>Disagree<br>Neither<br>Agree<br>Strongly Agree	0 (0%)<br>2 (22.2%)<br>4 (44.4%)<br>2 (22.2%)<br>1 (11.1%)
For each of the following statements, please indicate whether you agree or disagree: “My kids learned new information about water by using the app”	Strongly Disagree<br>Disagree<br>Neither<br>Agree<br>Strongly Agree	0 (0%)<br>2 (22.2%)<br>3 (33.3%)<br>3 (33.3%)<br>1 (11.1%)
For each of the following statements, please indicate whether you agree or disagree: “My kids learned new information about sugary drinks by using the app”	Strongly Disagree<br>Disagree<br>Neither<br>Agree<br>Strongly Agree	0 (0%)<br>2 (22.2%)<br>3 (33.3%)<br>3 (33.3%)<br>1 (11.1%)
For each of the following statements, please indicate whether you agree or disagree: “My kids learned more about what they are supposed to drink by using the app”	Strongly Disagree<br>Disagree<br>Neither<br>Agree<br>Strongly Agree	0 (0%)<br>2 (22.2%)<br>3 (33.3%)<br>3 (33.3%)<br>1 (11.1%)
For each of the following statements, please indicate whether you agree or disagree: “The app motivated my kids to want to change their drink choices”	Strongly Disagree<br>Disagree<br>Neither<br>Agree<br>Strongly Agree	0 (0%)<br>3 (33.3%)<br>3 (33.3%)<br>2 (22.2%)<br>1 (11.1%)
For each of the following statements, please indicate whether you agree or disagree: “My kids thought the app was fun”	Strongly Disagree<br>Disagree<br>Neither<br>Agree<br>Strongly Agree	0 (0%)<br>1 (11.1%)<br>3 (33.3%)<br>4 (44.4%)<br>1 (11.1%)
For each of the following statements, please indicate whether you agree or disagree: “My kids were able to understand most of the content on the app”	Strongly Disagree<br>Disagree<br>Neither<br>Agree<br>Strongly Agree	0 (0%)<br>2 (22.2%)<br>3 (33.3%)<br>4 (44.4%)<br>0 (0%)
For each of the following statements, please indicate whether you agree or disagree: “My kids would keep using this app in the future if they could”	Strongly Disagree<br>Disagree<br>Neither<br>Agree<br>Strongly Agree	0 (0%)<br>1 (11.1%)<br>3 (33.3%)<br>4 (44.4%)<br>0 (0%)
How often did other adults in your household use the app?	Never<br>A few times but not every day<br>Once a day<br>More than once a day<br>N/A	15 (68.2%)<br>2 (9.1%)<br>0 (0%)<br>0 (0%)<br>5 (22.7%)
Which would you say best describes how you/your family used the app over the past 6 months?	Frequently at first, less use at time went on<br>Rarely at first, more use at time went on<br>Frequently over 6 last mos<br>Rarely over last 6 mos	15 (68.2%)<br>2 (9.1%)<br>3 (13.6%)<br>2 (9.1%)

IVR Calls

Survey Question	Response Options	N (%) or Mean (sd)

Think about the automated phone calls that you received over the last 6 months. For each of the following statements about these calls, please indicate whether you agree or disagree: “I enjoyed getting the phone calls about drink choices for my family”	Strongly Disagree<br>Disagree<br>Neither<br>Agree<br>Strongly Agree	2 (8.3%)<br>1 (4.2%)<br>3 (12.5%)<br>15 (62.5%)<br>3 (12.5%)
Think about the automated phone calls that you received over the last 6 months. For each of the following statements about these calls, please indicate whether you agree or disagree: “Because of the phone calls, I learned new ways to get my family to drink more water”	Strongly Disagree<br>Disagree<br>Neither<br>Agree<br>Strongly Agree	0 (0%)<br>1 (4.2%)<br>6 (25%)<br>16 (66.7%)<br>1 (4.2%)
Think about the automated phone calls that you received over the last 6 months. For each of the following statements about these calls, please indicate whether you agree or disagree: “Because of the phone calls, I learned new ways to get my family to drink fewer sugary drinks”	Strongly Disagree<br>Disagree<br>Neither<br>Agree<br>Strongly Agree	1 (4.2%)<br>2 (8.3%)<br>5 (20.8%)<br>15 (62.5%)<br>1 (4.2%)
Think about the automated phone calls that you received over the last 6 months. For each of the following statements about these calls, please indicate whether you agree or disagree: “I set goals for myself or my child based on the phone calls”	Strongly Disagree<br>Disagree<br>Neither<br>Agree<br>Strongly Agree	0 (0%)<br>2 (8.3%)<br>6 (25%)<br>15 (62.5%)<br>1 (4.2%)
Think about the automated phone calls that you received over the last 6 months. For each of the following statements about these calls, please indicate whether you agree or disagree: “I was more comfortable with my family drinking tap water because of the phone calls”	Strongly Disagree<br>Disagree<br>Neither<br>Agree<br>Strongly Agree	1 (4.2%)<br>10 (41.7%)<br>5 (20.8%)<br>7 (29.2%)<br>1 (4.2%)
Think about the automated phone calls that you received over the last 6 months. For each of the following statements about these calls, please indicate whether you agree or disagree: “I would recommend these phone calls to other parents wanting to change their family's drink choice habits”	Strongly Disagree<br>Disagree<br>Neither<br>Agree<br>Strongly Agree	0 (0%)<br>4 (16.7%)<br>1 (4.2%)<br>16 (66.7%)<br>3 (12.5%)
Did you ever have any technical problems with the phone calls?	Voice speaking too quiet<br>Voice speaking too fast<br>Call did not register response<br>Call dropped<br>None of these problems	1 (4.2%)<br>1 (4.2%)<br>8 (33.3%)<br>4 (16.7%)<br>12 (50%)

Completion Rate of IVR Calls, By Call Number in Series

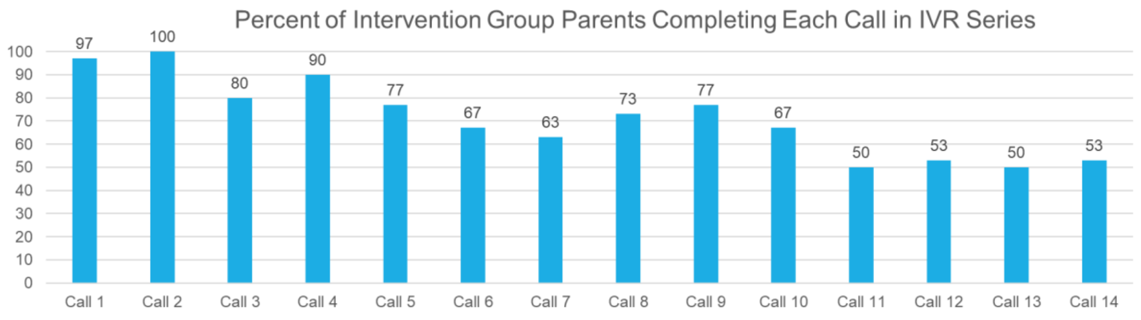

Supplement: Supplementary file 1 [file nutrients-15-02141-s001.zip › nutrients-2301753-supplementary.pdf]
